# The position of the Azeliinae in the Muscidae (Diptera) based on musculature of the male terminalia

**DOI:** 10.3897/zookeys.975.55502

**Published:** 2020-10-12

**Authors:** Vera S. Sorokina, Olga G. Ovtshinnikova

**Affiliations:** 1 Institute of Systematics and Ecology of Animals, Siberian Branch of the Russian Academy of Sciences, Novosibirsk, 630091, Russia Institute of Systematics and Ecology of Animals Novosibirsk Russia; 2 Zoological Institute, Russian Academy of Sciences, St. Petersburg, 199034, Russia Russian Academy of Sciences St. Petersburg Russia

**Keywords:** abdominal segments, Calyptratae, flies, male genitalia, muscles, Muscoidea, pregenital segments, sclerites

## Abstract

The male genital and pregenital skeleton and musculature were studied in males of the following species of the Muscidae subfamily Azeliinae: *Drymeia firthiana* (Huckett, 1965), *Drymeia longiseta* Sorokina & Pont, 2015, *Drymeia segnis* (Holmgren, 1883), *Thricops nigritellus* (Zetterstedt, 1838), *Thricops hirtulus* (Zetterstedt, 1838), *Hydrotaea dentipes* (Fabricius, 1805), *Muscina stabulans* (Fallén, 1817), and *Muscina levida* (Harris, 1780). Descriptions and figures of the genital sclerites and muscles of *D. firthiana* and *M. stabulans* are given. A comparison was made between the genital segments and muscles of previously studied species of Mydaeinae and Muscinae and those of the Azeliinae. Based on the structure of the skeleton and muscles of syntergosternite VII + VIII and the phallapodeme muscles, significant differences were found between the subfamily Azeliinae and the subfamilies Mydaeinae and Muscinae. The basal position of the Azeliinae within the family Muscidae was confirmed. A comparison of the genital segments and muscles of the Muscidae with those of the Scathophagidae (*Scathophaga stercoraria* (Linnaeus, 1758)) and Anthomyiidae (*Delia platura* (Meigen, 1826)) was made. Tendencies in reduction of the pregenital segments and musculature, as well as of the phallapodeme muscles in the evolution of the Muscoidea have been revealed. The complete set of phallapodeme muscles in the Scathophagidae and Anthomyiidae corresponds to the basal state, and therefore the structure of the genital sclerites and muscles in the Muscidae shows a certain degree of reduction. The progressive changes in the Muscidae from the Azeliinae through the Mydaeinae to the Muscinae were traced.

## Introduction

The Muscidae is one of the largest family of the Calyptratae (Diptera) and the largest of the Muscoidea. The world fauna includes approximately 5000 species in 180 genera (Pape et al. 2011). Despite the use of various modern methods of phylogenetic analysis, the classification of the family is still unstable and changeable, and sometimes controversial (Carvalho 1989; Carvalho et al. 2005; Schuehli et al. 2007; Fan 2008; Kutty et al. 2010, 2014, 2019; Haseyama et al. 2015; Grzywacz et al. 2017). The monophyly of the family and of some of its subfamilies (i.e., Azeliinae, Muscinae, and Coenosiinae) has been established beyond doubt, based on morphological characters and molecular data. However, the monophyly of some subfamilies (i.e., Mydaeinae and Phaoniinae) has not been supported and the traditional tribal classification has been rejected (Kutty et al. 2014, 2019; Haseyama et al. 2015).

Among the morphological characters used in phylogenetic reconstructions and classification systems, the characters describing the morphology of the muscles of the genital and pregenital structures are usually more stable than those of the sclerites (Matsuda 1976; Ovtshinnikova 1989; Friedrich and Beutel 2008). Moreover, study of the muscles helps to clarify function and homology and reveals parallelisms in the pregenital and genital sclerites (Ovtshinnikova 1989, 1994; Ovtshinnikova and Yeates 1998; Galinskaya and Ovtshinnikova 2015; Galinskaya et al. 2018; Ovtshinnikova et al. 2019).

Very few papers have dealt with the study of the male genital muscles of the Muscoidea. Hennig (1976) produced the first work on the Anthomyiidae, describing in detail the male genital muscles of *Delia platura* (Meigen, 1826) and *Fucellia tergina* (Zetterstedt, 1845), while the musculature of the pregenital sclerites was not studied. Later Ovtshinnikova (1989, 1994) studied the muscles in *Musca domestica* Linnaeus, 1758 and *Scathophaga stercoraria* (Linnaeus, 1758). Most recently, studies of the musculature of the male terminalia of the Muscoidea, and in particular that of the Muscidae, have continued with Ovtshinnikova et al. (2018, 2019) detailing the muscles of the genital and pregenital structures in some members of the Muscinae (*Musca autumnalis* De Geer, 1763, *Pyrellia rapax* (Harris, 1780)) and the Mydaeinae (*Mydaea urbana* (Meigen, 1826), *Graphomya maculata* (Scopoli, 1763)).

This paper continues the series of publications devoted to the structure of the sclerites and muscles of the abdominal segments and male terminalia in Muscidae and presents the results of our study of another subfamily, the Azeliinae. The Azeliinae was recognized as a subfamily following cladistic analyses by Carvalho (1989, 2002) and Carvalho et al. (2005). Previously, according the Hennig’s classification, the Azeliini was treated as a tribe within the subfamily Muscinae and this was followed by many authors (e.g., Hennig 1965; Pont 1986; Gregor at al. 2002). However, Skidmore (1985) maintained the Azeliinae and Reinwardtiinae as separate subfamilies mainly because the larvae of Azeliinae are trimorphic or dimorphic, facultative or obligate carnivores, or parasites, unlike the larvae of other Muscinae (Skidmore 1985). Before this study, Lobanov (1979, 1984) had written about a branch of the Azeliinae (“Hydrotaeinae” in Lobanov) on the basis of his studies of the female ovipositor. Savage and Wheeler (2004) conducted a genus-level phylogenetic analysis of the tribe Azeliini within the composition of the subfamily Azeliinae.

The subfamily Azeliinae is currently divided into two tribes: Azeliini and Reinwardtiini. Most of the members of the subfamily are known as anthophilous insects, but others are known as sweat flies or synanthropic flies. The larvae are mainly carnivores and can develop in humus soil, or are saprophages developing in various decomposing substrates including human and animal feces, or are even parasites of birds. The subfamily is cosmopolitan, but with a much higher diversity in the northern hemisphere for the Azeliini (389 species in 12 genera) and in the southern hemisphere for the Reinwardtiini (128 species in 17 genera) (A. C. Pont, pers. comm.).

The monophyly of the subfamily Azeliinae is still a matter for discussion. Only the monophyly of the tribe Azeliini has been established, based on morphological characters, molecular data and also the structure and lifestyle of the larval stage (Savage and Wheeler 2004; Schuehli et al. 2007; Kutty et al. 2014; Haseyama et al. 2015). The Azeliinae are paraphyletic in all molecular analyses because Muscinae is sister-group to the Azeliini while the Reinwardtiini are polyphyletic. There are no morphological synapomorphies to support monophyly of the current Azeliinae (Azeliini + Reinwardtiini), nor do the Reinwardtiini emerge as sister-group of the Azeliini, though according to other classifications the tribe Reinwardtiini has also been shown to be the sister-group of the Azeliini (Carvalho 2002; Savage and Wheeler 2004; Carvalho et al. 2005; Savage 2009). It has even been suggested that the Reinwardtiini should be treated as a separate subfamily (Skidmore 1985; Couri and Carvalho 2003). The monophyly of the Azeliinae has been confirmed in the recent paper by Kutty et al. (2019). However, only one species (*Muscina stabulans* Fallén, 1817) was used in this analysis.

This paper presents the results of our study of the sclerites and muscles of the male abdominal segments and terminalia in members of the subfamily Azeliinae belonging to the genera *Drymeia* Meigen, 1826, *Thricops* Rondani, 1856, *Hydrotaea* Robineau-Desvoidy, 1830 (Azeliini), and *Muscina* Robineau-Desvoidy, 1830 (Reinwardtiini).

## Materials and methods

The muscid material used in this paper is deposited in the collection of the Institute of Systematics and Ecology of Animals, Russian Academy of Sciences, Siberian Branch, Novosibirsk, Russia (**SZMN**).

To study the genital sclerites, dry specimens were softened in a hydration chamber; the abdomen was then detached, treated with 10% KOH solution, and dissected. The sclerites are designated here following the terminology of Sinclair (2000). The male abdomen consists of five segments; the pregenital segments VI–VIII are strongly modified as a result of the clockwise rotation of the male genitalia by 360°; the genital segments IX–XI are strongly modified.

The muscles of the male genitalia were studied by manual dissection of specimens preserved in 70% ethanol, using microknives, under a Leica MZ9^5^ stereomicroscope. The illustrations were made in Photoshop CS6 and CorelDRAW X6, based on digital images of muscles and sclerites captured with a Canon EOS 77D camera mounted on the Leica MZ9^5^ trinocular head. The genital muscles are classified into the following groups: abdominal, pregenital and genital muscles (tergosternal muscles, muscles of the hypandrial complex, and muscles of the epandrial complex). The muscles are numbered according to the classification of Ovtshinnikova (1989, 2000) and grouped by the sites of their origin.

The following abbreviations are used in the text: **c** – cercus; **dph** – distiphallus; **ej** – ejaculatory apodeme; **ep** – epandrium; **eph** – epiphallus; **hyp** – hypandrium; **l** – left muscle; **r** – right muscle; **pgt** – postgonite; **phap** – phallapodeme; **prgt** – pregonite; **sbeps** – subepandrial sclerite; **sp** – spiracle; **st** – sternite; **stgst** – syntergosternite; **sur** – surstylus; **tg** – tergite; **ISM** – abdominal and pregenital intersegmental sternal muscles; **ITM** – abdominal and pregenital intersegmental tergal muscles; **M1–M26** – pregenital and genital muscles; **TSM** – abdominal and pregenital tergosternal muscles.

The muscle **M18** includes asymmetric muscles which are designated in this paper as **M18 r** and **M18 l**. This corresponds to the previously accepted designations **M18^1^** and **M18^2^** in *Scathophaga* (Ovtshinnikova 1994).

Because of genital rotation, sclerites of the pregenital segments do not always lie in the usual position. For this reason, characteristics such as “wide” or “narrow” in the descriptions describe only the geometric shape of the sclerites, regardless of their orientation relative to the body axis.

## Results

### Muscidae, Azeliinae

The structure of the sclerites of the male terminalia has been previously studied and illustrated in 26 species of *Drymeia* (Sorokina and Pont 2015), in one species of *Hydrotaea* (Sorokina and Pont 2011), and in one species of *Thricops* (Vikhrev and Sorokina 2009). The structure of the sclerites of the male genitalia of most of the known *Thricops* have been studied and clearly illustrated by Savage (2003). In addition to this, genital structures have been studied but not illustrated for many other species of these genera.

In this paper, the structure of the sclerites and muscles of the male terminalia were studied in the following species of the subfamily Azeliinae: *Drymeia firthiana* (Huckett, 1965), *D. longiseta* Sorokina & Pont, 2015, *D. segnis* (Holmgren, 1883), *Hydrotaea dentipes* (Fabricius, 1805), *Thricops hirtulus* (Zetterstedt, 1838), *T. nigritellus* (Zetterstedt, 1838), *Muscina stabulans*, and *M. levida* (Harris, 1780).

### Sclerites and musculature of the male terminalia of Azeliinae

#### Azeliini

Since the genital skeleton and musculature in the examined species of *Drymeia*, *Thricops*, and *Hydrotaea* are very similar, the sclerites and muscles of only one species are described and illustrated here.

##### Drymeia firthiana

Taxon classificationAnimaliaDipteraMuscidae

(Huckett, 1965)

1FCD2321-A12A-5327-ACF1-9253F92FEF69

**Figure 1.**
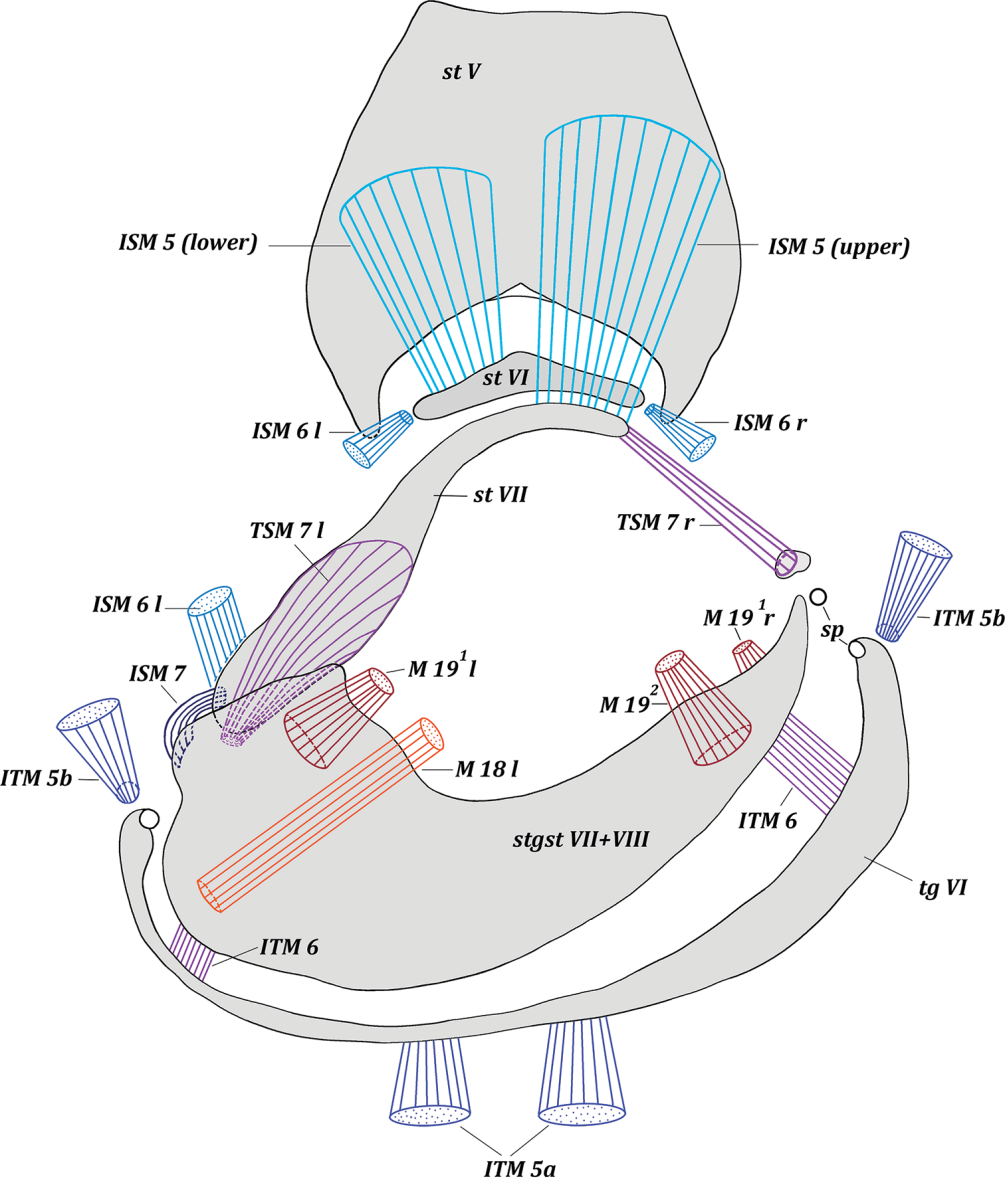
*Drymeia firthiana* (Huckett, 1965). Male pregenital segments, inner view. Upper musclesISM 5 removed left and lower musclesISM 5 removed right.

**Figure 2.**
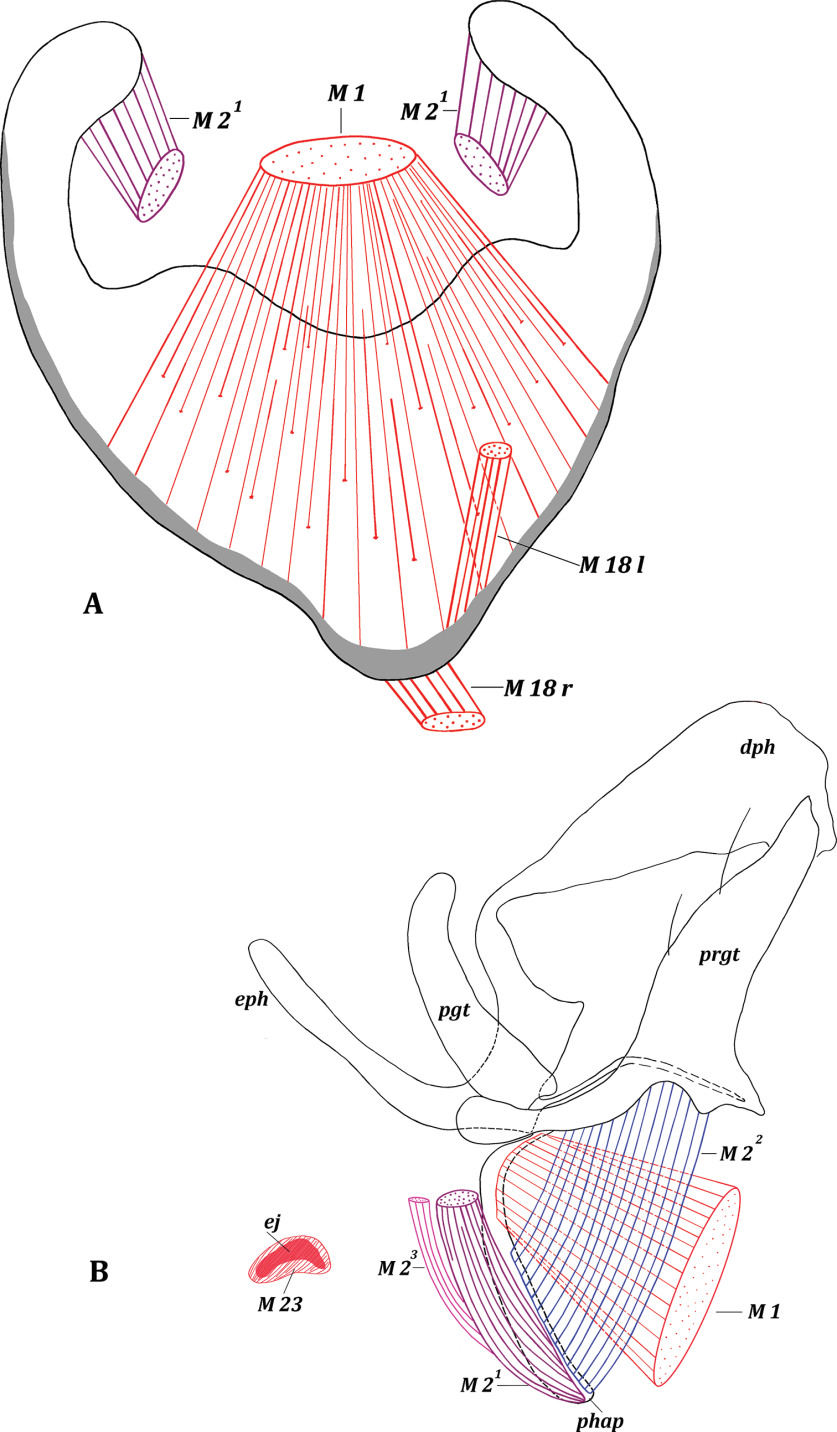
Male genitalia of *Drymeia firthiana* (Huckett, 1965) **A** hypandrium, inner view **B** aedeagal complex, lateral view.

**Figure 3.**
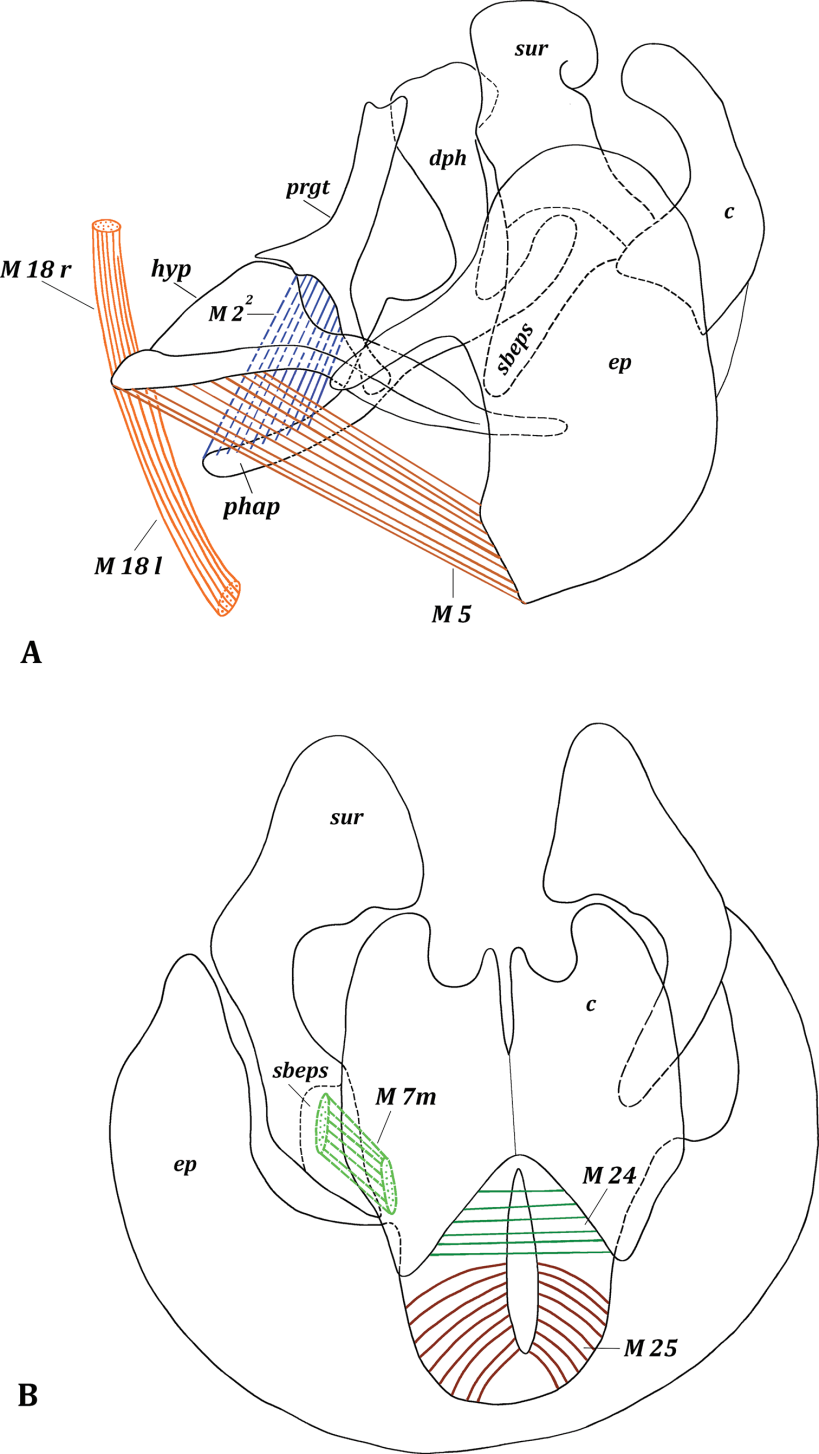
Male genitalia of *Drymeia firthiana* (Huckett, 1965) **A** genitalia, lateral view **B** epandrial complex, dorsal view.

**Figure 4.**
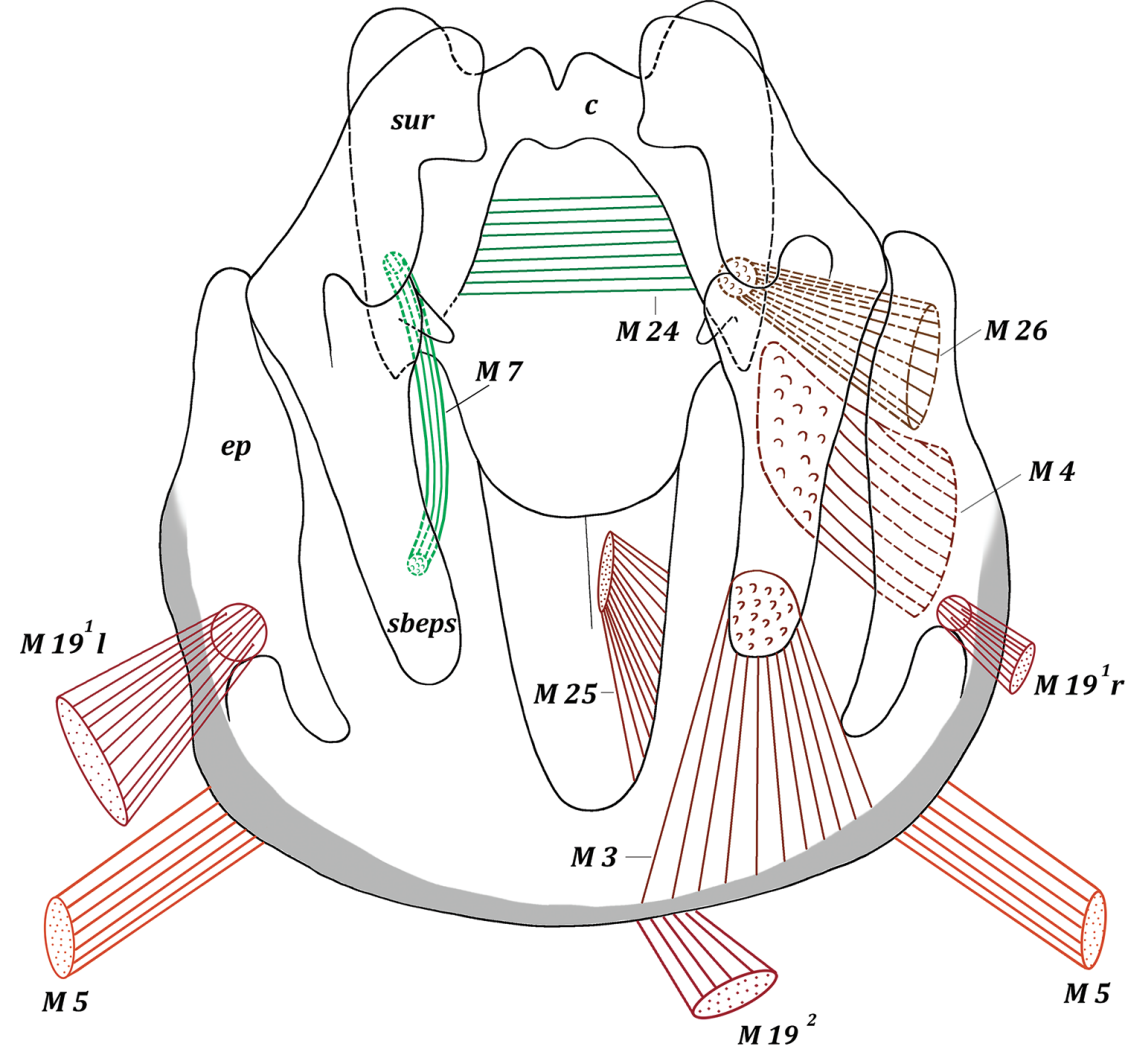
Male genitalia of *Drymeia firthiana* (Huckett, 1965). Epandrial complex, inner view.

Figures 1 , 2 , 3 , 4 , 9

###### Material examined.

10 males, Russia, Altai Republic, Kosh-Agach district, 8 km NE Maitobe Mt., 2420 m, 49°34'N, 87°43'E, pan traps, 7–10.vii.2006, leg. V. Sorokina.

###### Description.

***Abdominal segments*.** Sternite I reduced to narrow band, tergites I and II fused. Segments III and IV and tergite V not modified; sternite V enlarged, with wide median notch.

***Pregenital segments*** (Fig. 1). Tergite VI reduced to long narrow sclerotized band. Sternite VI positioned under sternite V, reduced in size, represented by short, narrow, wavy sclerite; remainder of sternite VI desclerotized. Sternite VII long, narrow, positioned on left side of body, dilated at articulation with syntergosternite VII + VIII; ventrally connected to desclerotized left margins of sternite VI; laterally connected to syntergosternite VII + VIII. Syntergosternite relatively wide, positioned dorsally; left end wider than right end and connected to sternite VII, right end free; posterior margin extended to epandrium.

***Genitalia*.** Hypandrium in form of concave plate, V-shaped (Fig. 2A); lateral arms of hypandrium articulated with surstyli and epandrium. Pregonites and postgonites present; pregonites larger than postgonites, tapered apically, and longer phallapodeme (Figs 2B, 9B). Phallus comprises epiphallus and distiphallus, basiphallus inconspicuous, either absent or fused with distiphallus. Phallapodeme long, articulated with phallus. Epiphallus well-developed, shaped as long and distally rounded plate. Distiphallus large, as long as pregonite, expanded distally, broadly articulated with epiphallus. Ejaculatory apodeme concave-shaped plate. Epandrium hemispherical, with large posteromedian notch (Fig. 3). Cerci large, wide, fused for a considerable length; each cercus with distal semicircular apical notch and well-formed process (Fig. 3B). Surstylus well-developed, wide, expanded and rounded apically, bent inward, with small process. Cercus approximately as long as surstylus. Subepandrial sclerite present as two long, quite wide, medially not connected plates, as long as length of surstyli and merging with them (Figs 4, 9F).

***Thoracic muscles***. Paired symmetrical conical muscles extend from thorax to lateromedian parts of tergite I + II, and also straight muscles extend from thorax to basal parts of sternite II.

***Abdominal muscles*** (Fig. 1): ITM 2–ITM 4, ITM 5a, ITM 5b, ISM 2–ISM 5, TSM 1–TSM 5. Flat, very short musclesITM 2–ITM 4 extend from distal parts of tergites II–IV along their entire width to basal margins of tergites III–V. Paired symmetrical musclesITM 5a extend from median parts of tergite V to median parts of basal margin of tergite VI. Long, paired, almost symmetrical conical musclesITM 5b extend from laterobasal parts of tergite V to membrane at lateral parts of tergite VI.

Paired symmetrical musclesISM 2–ISM 4 extend along the entire basal margin of sternites II–IV to basal margins of sternites III–V, respectively. Very powerful, paired, symmetrical, fan-shaped musclesISM 5 extend in two layers from basal margin of sternite V to sclerotized plate of sternite VI and to sternite VII at connection with membrane of sternite VI. Muscles extending to sternite VI connected with middle part of sternite V, but muscles extending to sternite VII connected with basal part of sternite V. Wide and flat pleural abdominal musclesTSM 1–TSM 5 easily discernible on corresponding segments.

***Pregenital muscles*** (Figs 1, 2A, 3A, 4): ITM 6, ISM 6, ISM 7, TSM 7, M 18, M 19^1^, M 19^2^. Small and short, paired, slightly asymmetrical musclesITM 6 extend from lateral parts of tergite VI to lateral parts of syntergosternite VII + VIII.

Paired musclesISM 6: left ISM 6 extends from left membranous parts of sternite VI to lateral margin of inner surface of sternite VII, articulated with syntergosternite VII + VIII; right muscle ISM 6 extends from right membranous parts of sternite VI to membrane near right laterobasal margin of syntergosternite VII + VIII; left ISM 6 larger than right muscle ISM 6. Unpaired left muscle ISM 7 short but powerful, extending from lateral margin of outer surface of sternite VII to outgrowth on lateral part of basal margin of syntergosternite VII + VIII. Paired asymmetrical musclesTSM 7: left muscle TSM 7 wide, short, fan-shaped, extending from lateral part of inner surface of sternite VII to small outgrowth on lateral margin of syntergosternite VII + VIII; right muscle TSM 7 long, fan-shaped, extending from right basal margin of sternite VII to small, sclerotized part of membrane adjacent to syntergosternite VII + VIII.

Paired asymmetrical muscles M 18: right muscle M 18 r wide and flat (homologous with left M 18 in Mydaeinae), extending from membrane covering genital cavity near syntergosternite VII + VIII to middle of basal margin of hypandrium (Figs 2A, 3A, 9C); left muscle M 18 l long (homologous with right M 18 in Mydaeinae), extending from left part of syntergosternite VII + VIII to inner surface of left laterobasal part of hypandrium. Paired asymmetrical muscles M 19^1^ (Fig. 4): left muscle M 19^1^ l short but powerful, extending from inner surface of small area on left lateral part of syntergosternite VII + VIII (close to connection with sternite VII) to small area on left laterobasal margin of epandrium; right muscle M 19^1^ r longer but weaker than M 19^1^ l, extending from right part of syntergosternite VII + VIII to right laterobasal margin of epandrium. Unpaired asymmetrical muscle M 19^2^ powerful, fan-shaped, and oblique, extending from right lateral part of syntergosternite VII + VIII to slightly to right from middle of basal margin of epandrium.

***Genital muscles*.***Tergosternal muscles* (Figs 3A, 9C): M 5. Paired, symmetrical powerful muscles M 5 extend from lateral parts of basal margin of hypandrium to median parts of basal margin of epandrium.

*Muscles of hypandrial complex* (Figs 2, 9A): M 1, M 2^1^, M 2^2^, M 2^3^, M 23. Wide and powerful, paired, symmetrical muscles M 1 extend from hypandrium, occupying considerable part of inner surface, to curve of mediobasal part of phallapodeme in front of pregonites. Long paired symmetrical muscles M 2^1^ extend from arms of hypandrium to laterodistal parts of phallapodeme. Powerful paired symmetrical muscles M 2^2^ extend from entire laterobasal part of pregonites to distal half of phallapodeme. Symmetrical muscles M 2^3^ long and close to each other, extending from membrane of basal margin of epiphallus between lateral ends of hypandrial arms to distal part of phallapodeme, opposite epandrium; muscles M 2^3^ very close to muscles M 2^1^ and both look like one muscle. Constrictors of ejaculatory apodeme small; muscles M 23 surrounding ejaculatory apodeme, contraction pumps seminal fluid into phallus.

*Muscles of epandrial complex* (Figs 3B, 4, 9E): M 3, M 4, M 7, M 24–M 26. Powerful paired symmetrical muscles M 3 extend from inner surface of basal parts of epandrium to inner surface of basal parts of subepandrial sclerite. Powerful paired symmetrical muscles M 4 extend from lateral parts of inner surface of epandrium to inner surface of basal parts of surstyli. Paired symmetrical thin cercal muscles M 7 extend from inner part of subepandrial sclerite to laterobasal parts of cerci. Broad powerful muscle M 24 passes inside cerci connecting lateral parts of two halves of cerci. Broad paired muscles M 25 extend from median parts of distal margin of epandrium to integument of anus. Powerful paired symmetrical, fan-shaped muscles M 26 extend from distolateral parts of epandrium to laterobasal margins of small cercal outgrowths.

##### Drymeia longiseta

Taxon classificationAnimaliaDipteraMuscidae

Sorokina & Pont, 2015

35F64A0D-7FC4-5B7F-87C0-6065F8007D09

###### Material examined.

8 males, Russia, Altai Republic, Kosh-Agach district, 8 km NE Maitobe Mt., 2420 m, 49°34'N, 87°43'E, pan traps, 7–10.vii.2006, leg. V. Sorokina.

###### Comment.

The muscles of this species are the same as *D. firthiana*.

##### Drymeia segnis

Taxon classificationAnimaliaDipteraMuscidae

(Holmgren, 1883)

2963F810-674C-5AA0-83FC-C6431DFFEF96

###### Material examined.

2 males, Russia, Krasnoyarsk Krai, Taymyr Peninsula, bank of River Zakharova Rassokha, 72°42'N, 101°06'E, in yellow pan traps, 11–20.vii.2011, leg. A. Barkalov.

###### Comment.

The muscles of this species and *D. firthiana* are the same.

##### Hydrotaea dentipes

Taxon classificationAnimaliaDipteraMuscidae

(Fabricius, 1805)

DE267339-6795-51FA-B093-F0A51BB571AB

###### Material examined.

3 males, Russia, Chukotka AO, 73 km W Anadyr, lower part of Anadyr River, 64°50'N, 175°58'E, 18–24.vii.2013, leg. A. Barkalov.

###### Comment.

The muscles of this speices and *D. firthiana* are the same.

##### Thricops hirtulus

Taxon classificationAnimaliaDipteraMuscidae

(Zetterstedt, 1838)

432A166C-35D5-5655-AA47-1F31EB15F713

###### Material examined.

2 males, Russia, Altai Republic, Kosh-Agach district, 8 km NE Maitobe Mt., 2420 m, 49°34'N, 87°43'E, pan traps, 7–10.vii.2006, leg. V. Sorokina.

###### Comment.

The muscles of this species and *D. firthiana* are the same.

##### Thricops nigritellus

Taxon classificationAnimaliaDipteraMuscidae

(Zetterstedt, 1838)

AA32DF88-1B46-5C80-8569-E788730DF3CE

###### Material examined.

5 males, Russia, Nenetz AO, Bolvanskaya Bay, pan traps, 68°05'N, 54°47'E, 18–25.vii.2015, leg. O. Makarova and M. Bizin.

###### Comment.

The muscles of this species and *D. firthiana* are the same.

#### Reinwardtiini

Among the Reinwardtiini, only *Muscina* is cosmopolitan, whilst the other 16 genera mostly occur in one or all of the tropical regions: Neotropical, Oriental, Australasian, and Afrotropical regions. One species each from *Passeromyia* Rodhain & Villeneuve, 1915 and *Synthesiomyia* Brauer & Bergenstamm, 1893 have also been found in the Palaearctic Region.

##### Muscina stabulans

Taxon classificationAnimaliaDipteraMuscidae

(Fallén, 1817)

4B1AAC3A-DD70-50C6-92DD-098A9C2AD6CA

**Figure 5.**
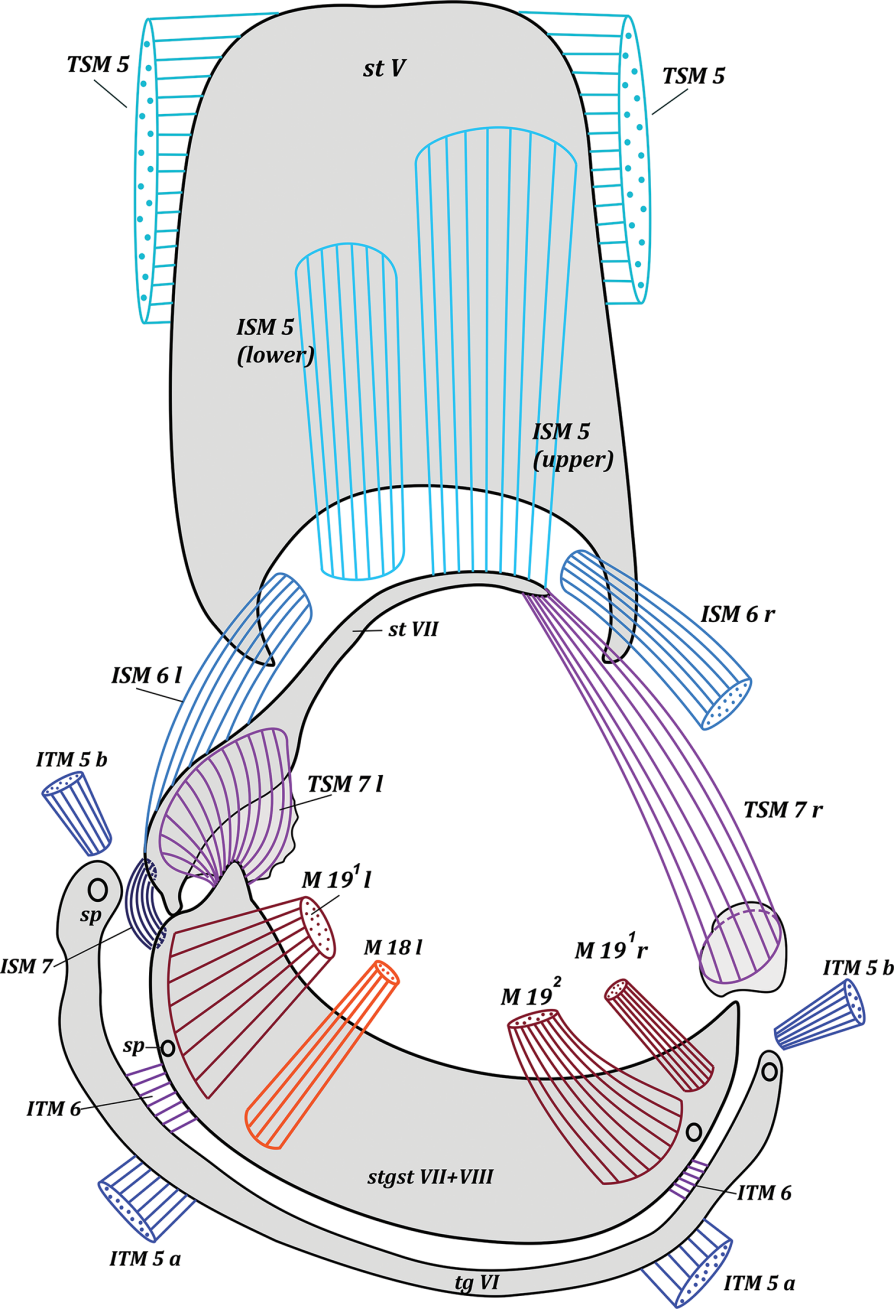
*Muscina stabulans* (Fallén, 1817). Male pregenital segments, inner view. Upper musclesISM 5 removed left and lower musclesISM 5 removed right.

**Figure 6.**
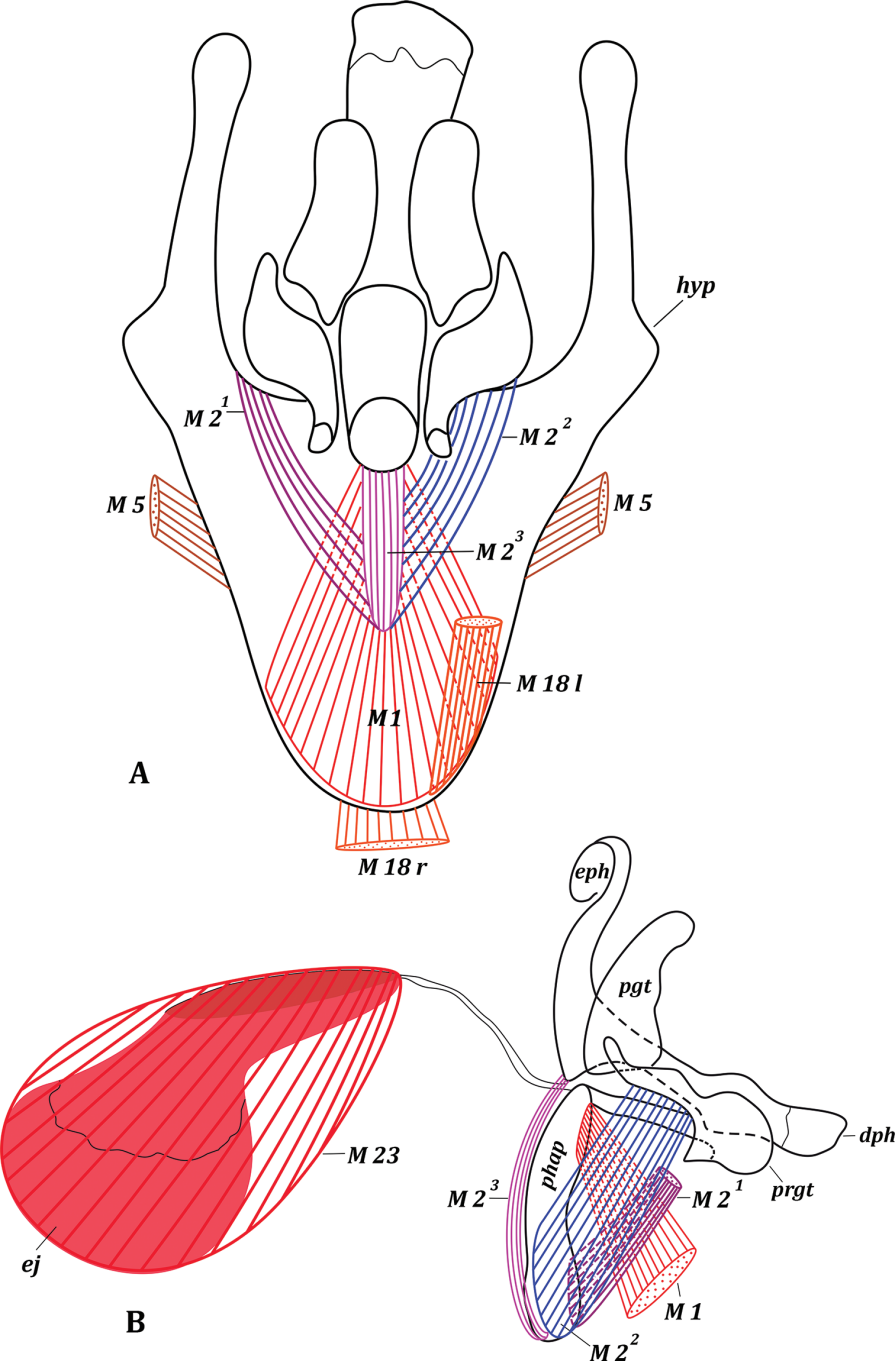
Male genitalia of *Muscina stabulans* (Fallén, 1817) **A** hypandrium, inner view **B** aedeagal complex, lateral view.

**Figure 7.**
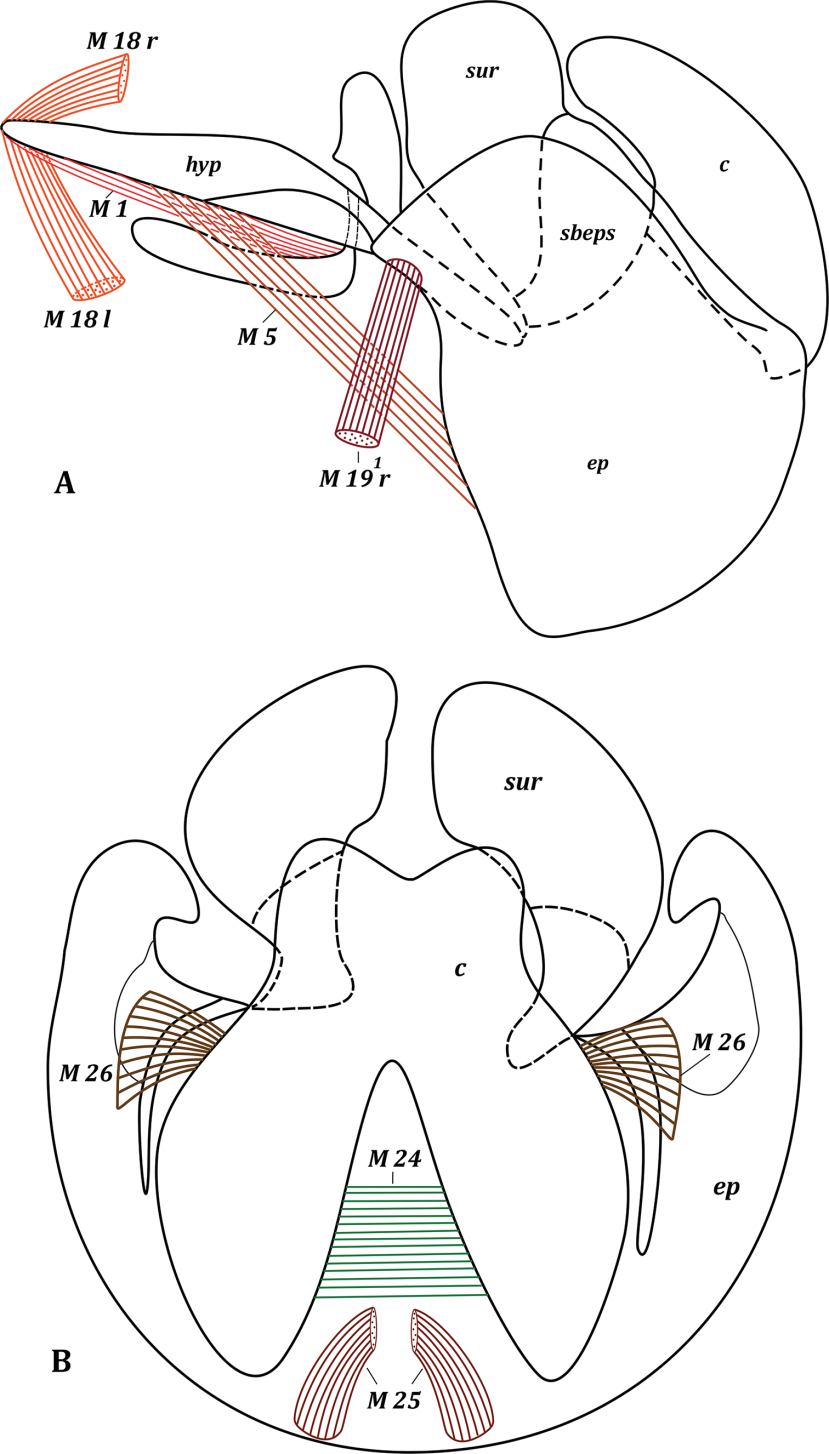
Male genitalia of *Muscina stabulans* (Fallén, 1817) **A** genitalia, lateral view **B** epandrial complex, dorsal view.

**Figure 8.**
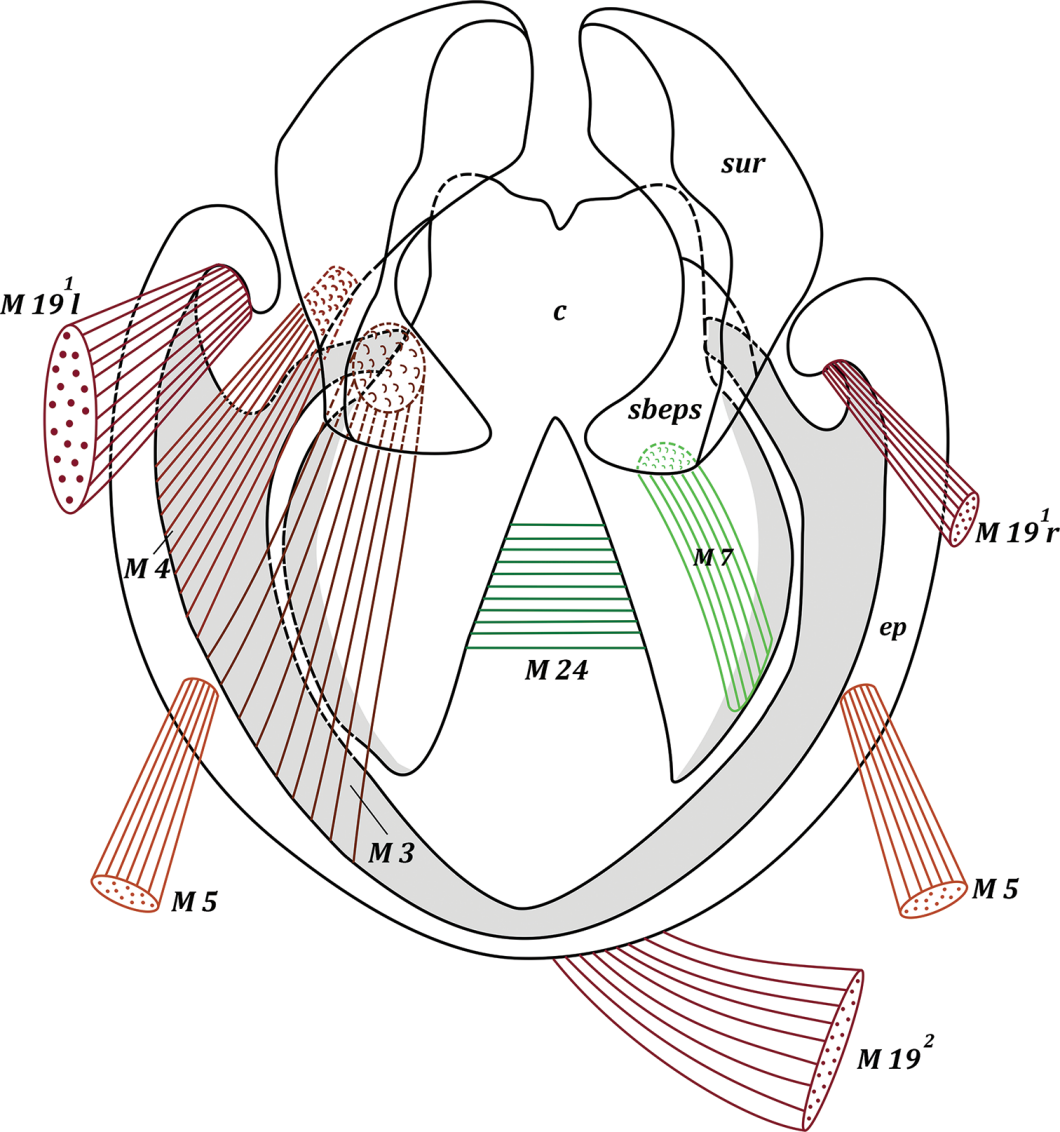
Male genitalia of *Muscina stabulans* (Fallén, 1817). Epandrial complex, inner view.

Figures 5 , 6 , 7 , 8

###### Material examined.

2 males, Russia, Kurgan region, Lebyazh’e district, environs of Lisje village, 55°08'N, 66°47'E, gardens, 15.vii.2019, leg. V. Sorokina. 4 males, Leningrad region, Vyborg district, Gorkovskoe railway station, Skiph, 60°17'N, 29°31'E, 1–7.viii.2018, leg. V. Sorokina.

###### Description.

***Abdominal segments*.** Sternite I reduced to narrow band, tergites I and II fused. Segments III and IV and tergite V not modified; sternite V enlarged, with wide median notch.

***Pregenital segments*** (Fig. 5). Tergite VI reduced to long narrow sclerotized band. Sternite VI completely membranous. Sternite VII long, narrow, positioned on left side of body, dilated at articulation with syntergosternite VII + VIII; ventrally terminates on membrane between sternites V and VII (desclerotized sternite VI), laterally connected to syntergosternite VII + VIII. Syntergosternite VII + VIII relatively wide, positioned dorsally; left end wider than right end and connected to sternite VII, right end free; posterior margin extending to epandrium.

***Genitalia*.** Hypandrium in form of concave plate, elongated, V-shaped (Fig. 6A); lateral arms of hypandrium articulated with surstyli and epandrium (Fig. 7A). Pregonites and postgonites of same size and both shorter than phallapodeme; pregonites tapered distally (Fig. 6B). Phallus containing epiphallus and distiphallus; basiphallus inconspicuous, either absent or fused with distiphallus. Phallapodeme long, articulated with phallus. Epiphallus well-developed, shaped as long, distally rounded plate. Distiphallus not large, as long as epiphallus, little expanded distally. Ejaculatory apodeme very large, sclerotized, plate-like, rounded apically (Fig. 6B). Epandrium semispherical, with large posteromedian notch (Figs 7B, 8). Cerci large, wide, fused distally (Fig. 7B). Surstylus well developed, wide, expanded and rounded apically, bent inwards, with small process. Subepandrial sclerite present as two short, quite wide, not medially connected plates, merging with surstyli (Fig. 8).

***Thoracic muscles***. Paired symmetrical conical muscles extend from thorax to lateromedian parts of tergite I + II, and also straight muscles extend from thorax to basal parts of sternite II.

***Abdominal muscles*** (Fig. 5): ITM 2–ITM 4, ITM 5a, ITM 5b, ISM 2–ISM 5, TSM 1–TSM 5. Flat, very short musclesITM 2–ITM 4 extend from distal parts of tergites II–IV along their entire width to basal margins of tergites III–V. Paired and slightly asymmetrical musclesITM 5a extend from median parts of tergite V to lateromedian parts of basal margin of tergite VI. Long, paired, slightly asymmetrical conical musclesITM 5b extend from laterobasal parts of tergite V to membrane at lateral parts of tergite VI.

Paired symmetrical musclesISM 2–ISM 4 extend along entire basal margin of sternites II–IV to basal margins of sternites III–V, respectively. Paired symmetrical musclesISM 5 extend in two layers from sternite V to membrane between sternite V and sternite VII (membranous sternite VI), spread along this membrane, and extend to sternite VII at connection with membrane of sternite VI (powerful, fan-shaped muscles). Muscles extending along membrane of sternite VI (lower layer) connected with distal part of sternite V, but muscles extending to sternite VII (upper layer) connected with basal part of sternite V. Wide and flat pleural abdominal musclesTSM 1–TSM 5 easily discernible on corresponding segments.

**Figure 9.**
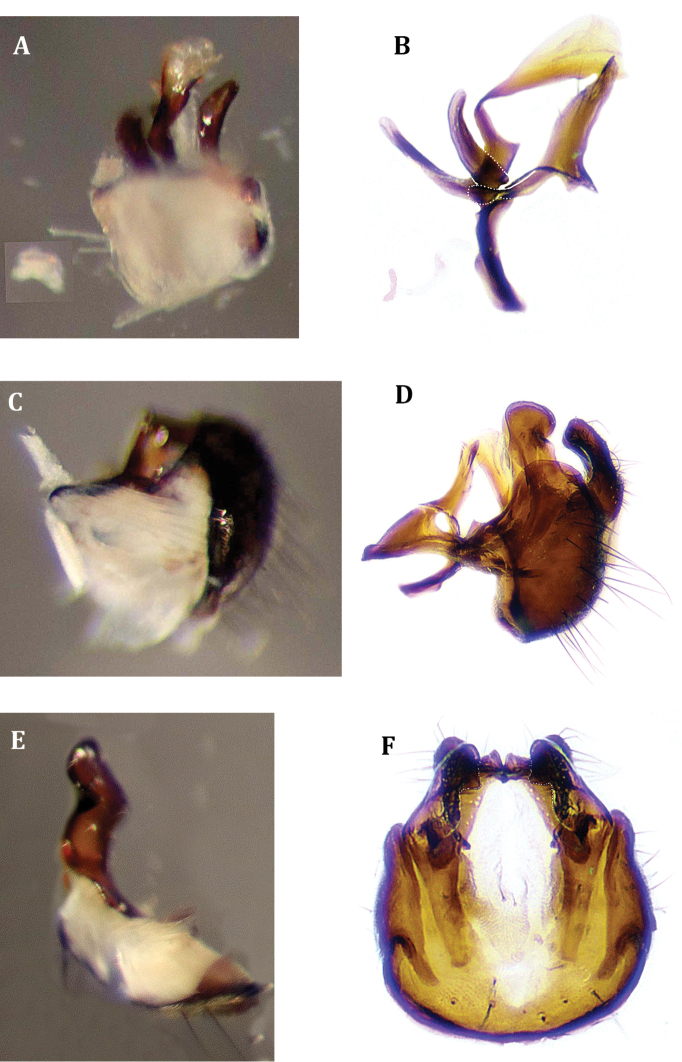
Male genitalia of *Drymeia firthiana* (Huckett, 1965) **A** aedeagal complex, lateral view, with muscles M 1, M 2^1^, M 2^2^, M 2^3^, M 23 and part of hypandrium, epiphallus removed **B** aedeagal complex, lateral view, sclerites **C** genitalia, lateral view, with muscles M 18 r, M 18 l and M 5 **D** genitalia, lateral view, sclerites **E** surstyli and subepandrial sclerite, inner view, with muscles M 4, M 3 **F** epandrial complex, inner view, sclerites.

***Pregenital muscles*** (Figs 5, 6A, 7A, 8): ITM 6, ISM 6, ISM 7, TSM 7, M 18, M 19^1^, M 19^2^. Small and short, paired, slightly asymmetrical musclesITM 6 extend from lateral parts of tergite VI to lateral parts of syntergosternite VII + VIII.

Paired musclesISM 6: left ISM 6 extends from left part of membrane of sternite VI to lateral margin of inner surface of sternite VII close to articulation with syntergosternite VII + VIII; right muscle ISM 6 extends from right parts of membrane of sternite VI to membrane near right laterobasal margin of syntergosternite VII + VIII; left ISM 6 larger than right muscle ISM 6. Unpaired left muscle ISM 7 short, powerful, extending from lateral margin of outer surface of sternite VII to outgrowth on lateral part of basal margin of syntergosternite VII + VIII. Paired asymmetrical musclesTSM 7: left muscle TSM 7 wide, short, fan-shaped, extending from lateral part of inner surface of sternite VII to small outgrowth on lateral margin of syntergosternite VII + VIII; right muscle TSM 7 fan-shaped, extending from right basal margin of sternite VII to small sclerite adjacent to syntergosternite VII + VIII.

Paired asymmetrical muscles M 18: right muscle M 18 r wide and flat, extending from membrane covering genital cavity near syntergosternite VII + VIII to middle of basal margin of hypandrium (Figs 6A, 7A); left muscle M 18 l long, extending from lateromedian left part of syntergosternite VII + VIII to inner surface of left laterobasal part of hypandrium. Paired asymmetrical muscles M 19^1^ (Fig. 8): left muscle M 19^1^ l powerful, extending from inner surface of left lateral part of syntergosternite VII + VIII (close to connection with sternite VII) to small area of left lateral margin of epandrium at connection with hypandrium; right muscle M 19^1^ r weaker than M 19^1^ l, extending from right part of syntergosternite VII + VIII to right lateral margin of epandrium at connection with hypandrium. Unpaired muscle M 19^2^ powerful, fan-shaped, and oblique, extending from right lateral part of syntergosternite VII + VIII to slightly to right from middle of basal margin of epandrium.

***Genital muscles*.***Tergosternal muscles* (Fig. 7A): M 5. Paired, symmetrical, powerful muscles M 5 extend from lateral parts of basal margin of hypandrium to lateral parts of basal margin of epandrium.

*Muscles of hypandrial complex* (Fig. 6): M 1, M 2^1^, M 2^2^, M 2^3^, M 23. Wide and powerful, paired, symmetrical muscles M 1 extend from hypandrium, occupying considerable part of inner surface, to basal part of phallapodeme in front of pregonites. Paired symmetrical muscles M 2^1^ extend from base of hypandrial arms to laterodistal parts of phallapodeme, opposite hypandrium. Long paired symmetrical muscles M 2^2^ extend almost from entire basal part of pregonites to distal half of phallapodeme, opposite hypandrium. Symmetrical muscles M 2^3^ long and close to each other, extending from membranous basal margin of epiphallus to distal part of phallapodeme, opposite epandrium.

Constrictors of ejaculatory apodeme wide and powerful; muscles M 23 surrounding ejaculatory apodeme and extending from rounded wide margin to tapered margin, contraction pumps seminal fluid into phallus.

*Muscles of epandrial complex* (Figs 7, 8): M 3, M 4, M 7, M 24–M 26. Powerful paired symmetrical muscles M 3 extend from inner surface of basal parts of epandrium to inner surface of subepandrial sclerite. Powerful paired symmetrical muscles M 4 extend from lateral parts of inner surface of epandrium to inner surface of basal parts of surstyli. Paired symmetrical short and thin cercal muscles M 7 extend from inner part of basal part of subepandrial sclerite to laterobasal parts of cerci. Broad powerful muscle M 24 passes inside cerci, connecting lateral parts of two halves of cerci. Broad paired muscles M 25 extend from median parts of epandrium to integument of anus. Powerful, fan-shaped, paired symmetrical muscles M 26 extend from distolateral parts of epandrium (more medially than M 4) to lateral cercal outgrowths.

##### Muscina levida

Taxon classificationAnimaliaDipteraMuscidae

(Harris, 1780)

7BD78B76-A911-5796-B499-7F0D8C5DE3C2

###### Material examined.

4 males, Russia, Leningrad region, Vyborg district, Gorkovskoe railway station, Skiph, 60°17'N, 29°31'E, 1–7.viii.2018, leg. V. Sorokina.

###### Comment.

The muscles of this species and *M. stabulans* are the same.

## Discussion and conclusions

In the Muscidae as well as in other Cyclorrhapha, both the sclerites and the muscles of abdominal segments VI–VIII and partly IX are asymmetrical as a result of the clockwise rotation of the male genitalia by 360°. The pregenital sclerites of segments VI–VIII are partly reduced, modified, and fused. In our previous studies we used the characteristic features of the musculature to clarify the homologies of some male pregenital sclerites in the Muscidae. The homologies of the pregenital sclerites in members of different subfamilies of the Muscidae, in particular the nature of tergite VI, sternites VI and VII, syntergosternite VII + VIII, and of the hypandrial appendages, was confirmed by analysis of the muscle connections (Ovtshinnikova et al. 2018, 2019). It was confirmed that syntergosternite VII + VIII in the Muscidae consists of tergites VII and VIII, and at least part of sternite VIII.

The results on the muscles of the Azeliinae and their connections with the sclerites support our previous conclusions about the presence and the order of certain genital sclerites: tergite VI – syntergosternite VII + VIII – epandrium (tergite IX); sternite VI – sternite VII – syntergosternite VII + VIII – hypandrium (sternite IX).

The structure of the sclerites of the terminal segments in the examined species of Azeliini (*Drymeia*, *Thricops*, and *Hydrotaea*) is very similar. The main differences are the shape and the degree of development of some sclerites, for example the extent of sclerotization of the connection point of the muscle TSM 7 r. Unlike other examined species of Azeliini, both *Hydrotaea ignava* (Harris, 1780) and *H. aenescens* (Wiedemann, 1830) have a very strongly sclerotized distiphallus, very small ejaculatory apodeme, and tergite VI divided into two sclerites.

The pregenital and genital musculature in all the examined Azeliini is also very similar. The main differences are in some features of the connection points of the muscles and in their development. In members of different genera, the muscle ISM 6 extending to sternite VII can connect with the lateral margin of sternite VI or the adjacent membrane with sternite VI (desclerotized part of sternite VI). In *H. dentipes*, the muscles of phallapodeme M 2^1^ extending from the hypandrial arms are much more developed than in *Drymeia* and *Thricops*.

The study of the sclerites and muscles of the terminal segments of the Reinwardtiini (*Muscina stabulans*, *M. levida*) has shown some differences from the Azeliini. Sternite VI is completely membranous. Nevertheless, as in the Azeliini, musclesISM 5 in *Muscina* extend in two layers, one above the other, from sternite V to the membrane between sternite V and sternite VII (membranous sternite VI) and to sternite VII at its connection with the membrane of sternite VI. However, muscles of the lower layer of the Reinwardtiini are less powerful and spread along the membrane. The joints of the phallapodeme muscles M 2^1^ are different between the Azeliini and the Reinwardtiini. In particular, in the Azeliini these muscles extend from the hypandrial arms close to the base of the epiphallus and they are opposite the epandrium, whereas in the Reinwardtiini M 2^1^ extend from the hypandrium, i.e. are on the other side of the phallapodeme. In addition, unlike the large pregonites and postgonites of the Azeliini these structures are smaller in the Reinwardtiini. Compared with other Muscidae previously examined by us, the ejaculatory apodeme in *M. stabulans* and *M. levida* is a very large sclerotized plate located inside syntergosternite VII + VIII and the wide and powerful constrictors of ejaculatory apodeme M 23 extend from one margin of this plate to the other. The same genitalic structures of the Reinwardtiini, in particular the small pregonites and postgonites, and the very large ejaculatory apodeme were described in the Neotropical genus *Callainireinwardtia* by Savage (2009). However, in the genus *Passeromyia* of the Reinwardtiini the ejaculatory apodeme is small, not enlarged, but pregonites and postgonites are also small (Pont 1974). Since the structures of the male genitalia in this tribe were studied in fragments and not for all genera, it is currently difficult to say how much the ejaculatory apodeme size is an important feature of the tribe. Some molecular data demonstrated the differences between the Azeliini and the Reinwardtiini where the Reinwardtiini is not sister-group of the Azeliini (Schuehli et al. 2007; Kutty et al. 2014; Haseyama et al. 2015). In these analyses the affinity between most genera of the Reinwardtiini and Cyrtoneurininae has been shown and only *Reinwardtia* has been related with the Azeliini. The authors of these works suggested that the Reinwardtiini are polyphyletic, but we cannot confirm or refute it in present work because only one genus was studied by us. Thus, further study of these structures as well as the genital and pregenital muscles of different genera of the current Reinwardtiini can confirm polyphyly of the Reinwardtiini and change the position of some genera in the Muscidae and probably this tribe itself.

In our earlier papers on the study of muscles in the subfamilies Muscinae and Mydaeinae, we suggested that the features of the genital musculature in Scathophagidae were basal (plesiomorphic) (Ovtshinnikova et al. 2018, 2019). In these papers, the reduction tendencies in the structure of the sclerites and the genital and pregenital musculature in the Muscinae as compared with the Mydaeinae, basically the pregenital sclerites and phallapodeme, were also found. In the Muscinae syntergosternite VII + VIII is narrow, while it is wider (less strongly reduced and membranous) in the Mydaeinae. Correspondingly, the pregenital muscles are paired and better developed in the Mydaeinae. In the Azeliinae as compared with the Muscinae and Mydaeinae, the pregenital sclerites as well as the pregenital muscles extending from syntergosternite VII + VIII to the epandrium (M 19) and to the hypandrium (M 18) are very well developed. In particular, members of the Azeliinae have three well-developed muscles M 19 and a long M 18, whereas in members of the other subfamilies only one pair of M 19 and a small M 18 were found. Since the set of the pregenital muscles is a stable feature compared with the sclerotization of sternite VI, the complete set of pregenital muscles, in addition to the presence of non-membranous sclerites (joint of the muscle TSM 7 r) close to syntergosternite VII + VIII (a demonstration of a lesser reduction of the pregenital sclerites), indicates the basal position of the Azeliinae in the Muscidae.

In the Muscidae, differences in the development of hypandrial appendages, parts of the phallus (epiphallus, distiphallus, basiphallus) and their musculature as a result of the reduction processes, as well as their homologies, were noted earlier (Ovtshinnikova et al. 2018, 2019). These appendages are of great importance in copulation. In the studied genera of the Azeliinae, the pregonites, postgonites, and epiphallus are well developed.

The main difference in the genital musculature of the Azeliinae (Reinwardtiini and Azeliini) compared with other subfamilies of the Muscidae previously examined by us is the presence of the same large set of the phallapodeme muscles, specifically four pairs of phallapodeme muscles: M 1, M 2^1^, M 2^2^, M 2^3^ (from the hypandrium, pregonites and the epiphallus). The set of the phallapodeme muscles in different subfamilies of the Muscidae is different, but in the Mydaeinae and Muscinae there are only two pairs of the muscle M 2: in the Muscinae M 2^1^, M 2^2^ (from the hypandrium and pregonites) and in the Mydaeinae M 2^2^, M 2^3^ (from pregonites and epiphallus). Since the set of phallapodeme muscles is a stable feature, the complete set of phallapodeme muscles (M 1, M 2^1^, M 2^2^, M 2^3^) in the Azeliinae (as in *Scathophaga*) is considered to be the basal state and confirms the basal position of the subfamily Azeliinae within the entire family Muscidae. Moreover, our results confirms well separated subfamily Azeliinae (Carvalho 1989; Carvalho et al. 2005; Savage and Wheeler 2004; Kutty et al. 2014) but refuted newly proposed classification with only three subfamilies (Haseyama et al. 2015) because the Azeliinae and the Muscinae have different set of genital and pregenital muscles and the structure of genital and pregenital segments and they cannot be in one subfamilies as authors suggested.

Comparison of the genital skeleton and muscles in the studied species of Muscidae with those of *Scathophaga stercoraria* has shown that males of most Muscidae as well as *S. stercoraria* possess well-developed pregonites, postgonites and epiphallus. However *Scathophaga* has a larger set of muscles of the hypandrial complex: 4 pairs of phallapodeme muscles M 1, M 2^1^, M 2^2^, M 2^3^ and the ejaculatory apodeme muscle M 23, muscles M 41 extending from the hypandrium to the basiphallus processes, muscles M 42 extending from the pregonites to the hypandrium and 2 pairs of tergosternal muscles M 5. Within the Muscidae, the set of phallapodeme muscles varies among the subfamilies, but members of all the genera we have studied possess only 1 pair of tergosternal muscles M 5, and lack muscles M 41 and M 42.

Comparison of the phallapodeme muscles of *Scathophaga* with those of *Delia platura* by Hennig (1976) has shown that *D. platura* has the same phallapodeme muscles as in *Scathophaga* (in Hennig: M 35 – 37, M 38, M 40, M 41) and one extra pair of muscles extending from the postgonites to the phallapodeme (in Hennig: M 39). In our opinion, the complete set of phallapodeme muscles in Scathophagidae and Anthomyiidae corresponds to the basal state, and the structure of the genital sclerites and muscles in Muscidae therefore reveals a certain degree of reduction. Our results about the relationships among studied families of the Calyptratae are congruent with the previous molecular hypotheses by Kutty et al. (2010, 2019).

The genital and pregenital modifications that we have detected in the Muscidae, in particular the reduction of pregenital sclerites and musculature, as well as the phallapodeme muscles, have thus allowed us to trace the progressive changes from the Azeliinae through the Mydaeinae to the Muscinae.

## Supplementary Material

XML Treatment for Drymeia firthiana

XML Treatment for Drymeia longiseta

XML Treatment for Drymeia segnis

XML Treatment for Hydrotaea dentipes

XML Treatment for Thricops hirtulus

XML Treatment for Thricops nigritellus

XML Treatment for Muscina stabulans

XML Treatment for Muscina levida

## References

[B1] Carvalho de CJB (1989) Classificação de Muscidae (Diptera): uma proposta através da análise cladística. Revista Brasileira de Zoologia 6(4): 627–648. 10.1590/S0101-81751989000400009

[B2] Carvalho de CJB (2002) Muscidae (Diptera) of the Neotropical Region: taxonomy. Editora Universidade Federal do Paraná, Curitiba, 287 pp.

[B3] Carvalho de CJB Couri MS Pont AC Pamplona D Lopes SM (2005) A catalogue of the Muscidae (Diptera) of the Neotropical Region. Zootaxa 860: 1–282. 10.11646/zootaxa.860.1.1

[B4] Couri MS Carvalho de CJB (2003) Systematic relations among *Philornis* Meinert, *Passeromyia* Rodhain and Villeneuve and allied genera (Diptera, Muscidae). Brazilian Journal of Biology 63: 223–232. 10.1590/S1519-69842003000200007 14509844

[B5] Fan Z (2008) Fauna Sinica Insecta. DipteraMuscidae (1) (Vol. 49). Science Press, Beijing, 1180 pp.

[B6] Friedrich F Beutel RG (2008) The thorax of *Zorotypus* (Hexapoda, Zoraptera) and a new nomenclature for the musculature of Neoptera. Arthropod Structure & Development 37: 29–54. 10.1016/j.asd.2007.04.003 18089126

[B7] Galinskaya TV Ovtshinnikova OG (2015) Musculature of the male genitalia in *Rivellia* (Diptera: Platystomatidae). ZooKeys 545: 149–158. 10.3897/zookeys.545.6702 PMC4714374 26798301

[B8] Galinskaya TV Gafurova D Ovtshinnikova OG (2018) X-ray microtomography (microCT) of male genitalia of *Nothybus kuznetsovorum* (Nothybidae) and *Cothornobata* sp. (Micropezidae). ZooKeys 744: 139–147. 10.3897/zookeys.744.22347 PMC5904365 29670447

[B9] Gregor F Rozkošný R Barták M Vaňhara J (2002) The Muscidae (Diptera) of Central Europe. Folia Facultatis Scientiarum Naturalium Universitatis Masarykianae Brunensis, Biologia 107: 1–280.

[B10] Grzywacz A Wallman JF Piwczyński M (2017) To be or not to be a valid genus: the systematic position of *Ophyra* R.-D. revised (Diptera: Muscidae). Systematic Entomology 42: 714–723. 10.1111/syen.12240

[B11] Haseyama KLF Wiegmann BM Almeida EAB Carvalho de CJB (2015) Say goodbye to tribes in the new house fly classification: a new molecular phylogenetic analysis and an updated biogeographical narrative for the Muscidae (Diptera). Molecular Phylogenetics and Evolution 89: 1–12. 10.1016/j.ympev.2015.04.006 25869937

[B12] Hennig W (1965) Vorarbeiten zu einem phylogenetischen System der Muscidae (Diptera: Cyclorrhapha). Stuttgarter Beiträge zur Naturkunde 141: 1–100.

[B13] Hennig W (1976) 63a. Anthomyiidae [part]. In: Lindner E (Eds) Die Fliegen der Palaearktischen Region 7(1). Schweizerbart, Stuttgart, 78 pp.

[B14] Kutty SN Pape T Wiegmann BM Meier R (2010) Molecular phylogeny of the Calyptratae (Diptera: Cyclorrhapha) with an emphasis on the superfamily Oestroidea and the position of Mystacinobiidae and McAlpine’s fly. Systematic Entomology 35: 614–635. 10.1111/j.1365-3113.2010.00536.x

[B15] Kutty SN Pont AC Meier R Pape T (2014) Complete tribal sampling reveals basal split in Muscidae (Diptera), confirms saprophagy as ancestral feeding mode, and reveals an evolutionary correlation between instar numbers and carnivory. Molecular Phylogenetics and Evolution 78: 349–364. 10.1016/j.ympev.2014.05.027 24910153

[B16] Kutty SN Meusemann K Bayless KM Marinho MAT Pont AC Zhou X Misof B Wiegmann BM Yeates D Cerretti P Meier R Pape T (2019) Phylogenomic analysis of Calyptratae: resolving a major radiation of Diptera. Cladistics 35(6): 605–622. 10.1111/cla.12375 34618931

[B17] Lobanov AM (1979) Morphology, systematics and ecology of the family Muscidae (Diptera, Calyptrata). PhD thesis, Leningrad: Zoological Institute, Academy of Sciences of USSR.

[B18] Lobanov AM (1984) To the problem of the Muscidae phylogeny. Problems of Diptera evolution and phylogeny. Moscow, 5–17. [In Russian]

[B19] Matsuda R (1976) Morphology and Evolution of the Insect Abdomen. Headington Hill Hall Oxford England, Pergamon Press Ltd, 534 pp.

[B20] Ovtshinnikova OG (1989) Muscles of the male genitalia of Brachycera-Orthorrhapha (Diptera). Trudy Zoologicheskogo Instituta Akademii Nauk SSSR 190: 1–166.

[B21] Ovtshinnikova OG (1994) On the homology of male genital sclerites of BrachyceraOrthorrhapha and Cyclorrhapha (Diptera) based on musculature. Dipterological Research 5(4): 263–269.

[B22] Ovtshinnikova OG (2000) Muscles of the Male Genitalia of Syrphidae (Diptera). NA Kholodkovsky Memorial Lectures: a Report to the 52nd Annual Meeting, April 1, 1999. St. Petersburg, 70 pp. [in Russian]

[B23] Ovtshinnikova OG Galinskaya TV Sorokina VS (2018) Musculature of the male abdominal segments and terminalia in *Musca autumnalis* De Geer, 1776 and *Pyrellia rapax* (Harris, 1780) (Diptera, Muscidae: Muscini). Entomological Review 98(6): 678–689. 10.1134/S0013873818060040

[B24] Ovtshinnikova OG Sorokina VS Galinskaya TV (2019) Musculature of the male abdominal segments and terminalia of *Mydaea urbana* (Meigen, 1826) and *Graphomya maculata* (Scopoli, 1763) (Diptera, Muscidae: Mydaeinae). Entomological Review 99(5): 628–638. 10.1134/S0013873819050063

[B25] Ovtshinnikova OG Yeates DK (1998) Male genital musculature of Therevidae and Scenopinidae (Diptera: Asiloidea): structure, homology and phylogenetic implications. Australian Journal of Entomology 37(1): 27–33. 10.1111/j.1440-6055.1998.tb01539.x

[B26] Pape T Blagoderov V Mostovski MB (2011) Order Diptera Linnaeus, 1758. In: Zhang Z-Q (Eds) Animal biodiversity. An outline of higher-level classification and survey of taxonomic richness.Zootaxa 3148: 222–229. 10.11646/zootaxa.3148.1.42 26146682

[B27] Pont AC (1974) Revision of the genus Passeromyia Rodhain and Villeneuve (Diptera: Muscidae). Bulletin of British Museum Natural History Entomology 30: 339–372. 10.5962/bhl.part.24943

[B28] Pont AC (1986) Family Muscidae. In: Soós A Papp L (Eds) Catalogue of the Palearctic Diptera (Vol.11). Budapest, Akadémiai Kiadó, 57–215.

[B29] Savage J (2003) Revision of the genus *Thricops* Rondani (Diptera: Muscidae). Insect Systematics and Evolution Supplement 61: 1–143.

[B30] Savage J (2009) A new genus and new species of Neotropical Reinwardtiini (Diptera: Muscidae). Annals of the Entomological Society of America 102(3): 354–359. 10.1603/008.102.0302

[B31] Savage J Wheeler TA (2004) Phylogeny of the Azeliini (Diptera: Muscidae). Studia Dipterologica 11(1): 259–299.

[B32] Schuehli GS Carvalho de CJB Wiegmann BM (2007) Molecular phylogenetics of the Muscidae (Diptera: Calyptratae): new ideas in a congruence context. Invertebrate Systematics 21: 263–278. 10.1071/IS06026

[B33] Sinclair BJ (2000) 1.2. Morphology and terminology of Diptera male terminalia. In: Papp L Darvas B (Eds) Contributions to a Manual of Palaearctic Diptera (Vol.1). Science Herald, Budapest, 53–74.

[B34] Skidmore P (1985) The biology of the Muscidae of the world. Series Entomologica 29: 1–550.

[B35] Sorokina VS Pont AC (2011) Fanniidae and Muscidae (Insecta, Diptera) associated with burrows of the Altai Mountains Marmot (*Marmota baibacina baibacina* Kastschenko, 1899) in Siberia, with the description of new species. Zootaxa 3118: 31–44. 10.11646/zootaxa.3118.1.2

[B36] Sorokina VS Pont AC (2015) A review of the genus *Drymeia* Meigen, 1826 (Diptera: Muscidae) in Russia. Zootaxa 4000(2): 151–206. 10.11646/zootaxa.4000.2.1 26623610

[B37] Vikhrev NE Sorokina VS (2009) Faunistic records of *Thricops* Rondani (Diptera, Muscidae) from Russia with description of two new species. Euroasian Entomological Journal 8(3): 341–350.

